# Trichomes related to an unusual method of water retention and protection of the stem apex in an arid zone perennial species

**DOI:** 10.1093/aobpla/plu088

**Published:** 2014-12-19

**Authors:** Makeli Garibotti Lusa, Elaine Cristina Cardoso, Silvia Rodrigues Machado, Beatriz Appezzato-da-Glória

**Affiliations:** 1 Departamento de Ciências Biológicas, Escola Superior de Agricultura ‘Luiz de Queiroz’, Universidade de São Paulo, Piracicaba, São Paulo 13418-900, Brazil; 2 Programa de Pós-graduação em Biologia Vegetal, Instituto de Biologia, Universidade Estadual de Campinas, CP 6109, Campinas, São Paulo 13083-970, Brazil; 3 Departamento de Botânica, Instituto de Biociências, Universidade Estadual Paulista, Botucatu, São Paulo 18618-000, Brazil

**Keywords:** Asteraceae, cell-wall degradation, dehydration protection, glandular trichomes, histochemistry, non-glandular trichomes.

## Abstract

It is well known that trichomes protect plant organs, and several studies have investigated their role in the adaptation of plants to harsh environments. Recent studies have shown that the production of hydrophilic substances by glandular trichomes and the deposition of this secretion on young organs may facilitate water retention, thus preventing desiccation and favouring organ growth until the plant develops other protective mechanisms. *Lychnophora diamantinana* is a species endemic to the Brazilian ‘campos rupestres’ (rocky fields), a region characterized by intense solar radiation and water deficits. This study sought to investigate trichomes and the origin of the substances observed on the stem apices of *L. diamantinana.* Samples of stem apices, young and expanded leaves were studied using standard techniques, including light microscopy and scanning and transmission electron microscopy. Histochemical tests were used to identify the major groups of metabolites present in the trichomes and the hyaline material deposited on the apices. Non-glandular trichomes and glandular trichomes were observed. The material deposited on the stem apices was hyaline, highly hydrophilic and viscous. This hyaline material primarily consists of carbohydrates that result from the partial degradation of the cell wall of uniseriate trichomes. This degradation occurs at the same time that glandular trichomes secrete terpenoids, phenolic compounds and proteins. These results suggest that the non-glandular trichomes on the leaves of *L. diamantinana* help protect the young organ, particularly against desiccation, by deposition of highly hydrated substances on the apices. Furthermore, the secretion of glandular trichomes probably repels herbivore and pathogen attacks.

## Abstract (precis)

Plant adaptation strategies to harsh environments are among the most interesting subjects in plant biology. Several studies have investigated the role trichomes play in protecting plant organs in these conditions. In this work we report an unusual way of protecting the stem apex of *Lychnophora diamantinana*. The terminal cell of its non-glandular trichomes undergoes partial degradation of the cell wall, producing a highly hydrated, hyaline material that protects the stem apex against desiccation.

## Introduction

In nature, the trichomes can exhibit an enormous diversity in relation to the morphology, origin, size, location, timing of activity and function; often the trichomes are distinguished as glandular and non-glandular, according to their functions, mainly the capability to secrete (Werker 2000). It is well known that trichomes protect plant organs, and several studies have investigated their role in the adaptation of plants to harsh environments (Fahn 1979; Werker 2000). In the stem apices, the trichomes are commonly related to the protection of the young organs: the glandular trichomes release repellent compounds against herbivores and pathogens (Werker 2000; Siebert 2004; Göpfert *et al.* 2005; Machado *et al.* 2006) or also produce hydrophilic substances related to protection against desiccation (Bruni *et al.* 1987; Fahn 1990; Paiva and Martins 2011); and non-glandular trichome acting as a mechanical barrier against excessive light, water loss and extreme temperatures (Fahn and Cutler 1992; Turner 1994; Werker 2000).

Traditionally, colleters are secretory structures (emergences or glandular trichomes) more known by production of substances hydrophilic (mucilaginous or resinous), usually found on young organs (Fahn 1990; Paiva and Machado 2006; Evert 2007; Paiva 2009). Recent studies (Mayer *et al.* 2011; Paiva and Martins 2011; Cardoso-Gustavson *et al*. 2014) have shown the functional role of glandular trichomes producing hydrophilic substances on young organs preventing desiccation, maintaining the water status and favouring organ growth. However, more studies on the structure and function of non-glandular trichomes, or covering trichomes are necessary to understand their complete adaptive significance in young organs of plants that are exposed to intense solar radiation and drought.

*Lychnophora diamantinana* belongs to Lychnophorinae, a subtribe of Vernonieae (Asteraceae) almost restrict to Cerrado domain in Brazil; the species is endemic to the ‘campos rupestres’ and is found on rocky outcrops or slopes (Coile and Jones 1981). The ‘campos rupestres’ are vegetation formations in the Cerrado domain and are characterized by rocky formations with altitudes that are usually greater than 900 m; the soils are shallow, with rapid drainage, acidic and nutrient-poor, with low organic matter content, and originated from the decomposition of quartzite and arenite (Ribeiro and Walter 2008). These environments experience a dry season and a rainy season, and constant winds, intense solar radiation, daily temperature fluctuations and fires (during the dry season) are common (Goodland and Ferri 1979; Ratter *et al.* 1997; Simon *et al.* 2009). In this environment, the growth and development of new plant organs usually begins in the spring, before the rainy season (Ribeiro and Walter 2008), and thus, the plant will likely face extreme weather conditions that require the use of mechanisms to protect these organs. According to Appezzato-da-Glória and Estelita (2000), the substances rich in polysaccharides produced in shoot apices can prevent water loss in hot tropical climates, as seen in the Cerrado domain.

In this study, we describe for the first time an unusual way for water retention on the stem apices of *L. diamantinana* by the partial degradation of the cell wall of the terminal cells in the non-glandular trichomes. We also identify the origin and structure of the non-glandular trichomes and the secretion of the glandular trichomes, which we consider from a functional perspective.

## Methods

### Botanical materials

*Lychnophora diamantinana* is distributed along the Espinhaço Range in the State of Minas Gerais and is found at altitudes between 1300 and 1500 m (Coile and Jones 1981). The analysed material was collected in the Biribiri State Park, which is in the Municipality of Diamantina, Minas Gerais, Brazil. Voucher specimens were deposited in the SPF Herbarium (University of São Paulo) under voucher Loeuille *et al.* 530. Stem apices with leaf primordia, young leaves and completely expanded leaves were collected from three adult individuals, from August to November, during the transition period from the dry to the rainy season.

### Light microscopy

For light microscopy analyses, entire stem apices, entire leaf primordia and the middle third of young and mature leaves were fixed in Karnovsky's solution (Karnovsky 1965, modified using pH 7.2 phosphate buffer), placed in a vacuum chamber to remove the air in the tissues, dehydrated in an ethanol series and embedded in hydroxyethyl methacrylate Leica Historesin^®^ (Heraeus-Kulzer, Hanau, Germany), following the manufacturer's instructions, and sectioned at 5–7 µm thickness on a rotary microtome (Model RM 2245, Leica Microsystems Nussloch GmbH, Nussloch, Germany). For structural analysis, the sections were stained with toluidine blue 0.05 % in citrate–phosphate buffer, pH 4.5 (Sakai 1973), and mounted in synthetic resin Entellan^®^ (Merck^®^, Darmstadt, Germany).

### Histochemistry

Histochemical reactions were performed using fresh material, including the hyaline substance, and/or embedded material, as described above. The staining reactions included periodic acid-Schiff (PAS) for total polysaccharides (McManus 1948); calcofluor white M2R (Hughes and McCully 1975) for cellulose measurements using fluorescence; coriphosphine (Ueda and Yoshioka 1976) for pectin measurements using fluorescence; Sudan IV (Jensen 1962) for lipophilic substances; NADI reagent (David and Carde 1964) for terpenoids; ferric chloride (Johansen 1940) for general phenolic compounds; and aniline blue black (Fisher 1968) and xylidine ponceau (Vidal 1970) for proteins. The sections were examined immediately after each reaction. For the calcofluor white test, the sections were observed under a epifluorescence microscope (Model DM LB, Leica Microsystems Wetzlar GmbH, Wetzlar, Germany) equipped with an ‘HBO 100 W mercury vapour lamp’ and a violet excitation filter (bandpass filter; 355–425 nm). For the coriphosphine test, the induced fluorescence was observed using the same microscope with a blue excitation filter (bandpass filter; 420–490 nm). Control sections were examined simultaneously with the histochemical tests, using standard procedures. To determine the natural characteristics of the organs and secretions, untreated sections were mounted and observed. Light microscopy results were recorded using a video camera (DC 300F Leica Microsystems (Schweiz) AG, Heerbrugg, Switzerland) coupled to the Leica^®^ DM LB microscope to obtain images of the sections.

### Scanning electron microscopy

For scanning electron microscopy (SEM) analyses, samples were fixed in Karnovsky (Karnovsky 1965) solution for 24 h, dehydrated in a graded ethanol series and critical-point dried with CO_2_. The samples were attached to aluminium stubs and coated with gold (30–40 nm). Finally, the samples were examined under a LEO VP435 (Zeiss, Oberkochen, Germany) scanning electron microscope at 20 kV.

### Transmission electron microscopy

Transmission electron microscopy (TEM) was used for ultrastructure analysis. Samples of entire leaf primordia (2–5-mm-long) were collected with tweezers and fixed in glutaraldehyde (2.5 % in 0.1 M, pH 7.3, sodium phosphate buffer) for 24 h, post-fixed with osmium tetroxide (1.0 % in 0.1 M, pH 7.3, sodium phosphate buffer) for 2 hours and incubated in uranyl acetate (0.5 % in aqueous solution). The samples were dehydrated through a graded series of acetone and the material was embedded in Araldite resin. Ultrathin sections were contrasted using uranyl acetate and lead citrate (Reynolds 1963) and were examined under a Philips Model EM 100 transmission electron microscope operating at 80 kV.

## Results

**Figure 1.**
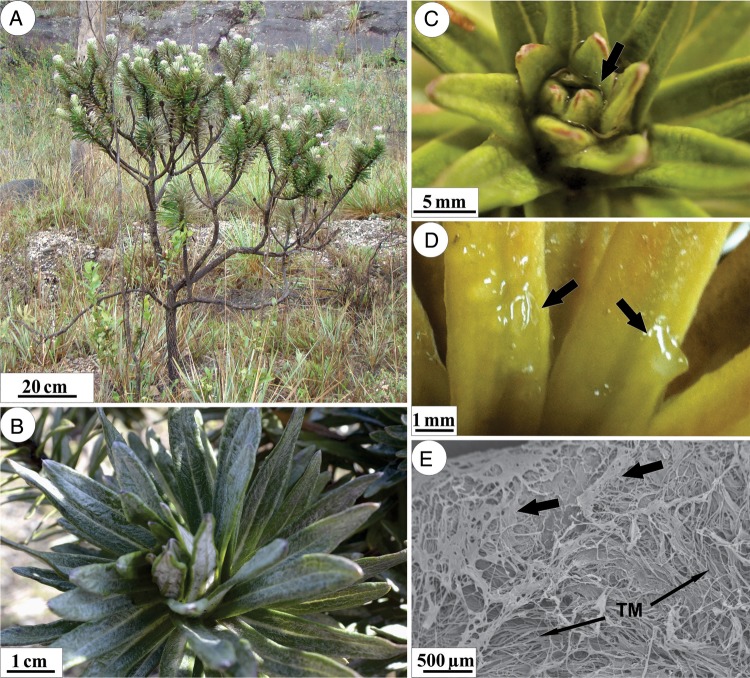
Morphological characteristics of *L. diamantinana*. (A) Treelet habit, exhibiting branches with overlapping leaves. (B) Stem apex with pronounced leaf overlap and whitish appearance. (C and D) Details of the apical region of the branch with hyaline substance (arrows) on the young leaves. (E) Scanning electron micrograph showing this substance (arrow) spread over and between the trichome mesh (TM) on the young leaf.

*Lychnophora diamantinana* is a treelet that has stems with very short internodes and overlapping leaves, spirally arranged (Fig. 1A and B). The leaf primordia and young leaves at the stem apices are covered by a viscous and hyaline substance (Fig. 1C and D), which is macroscopically visible in the field, especially after rain or fog. Young leaves are completely covered with the substance (Fig. 1C and D) that is spread over and between the trichome mesh (Fig. 1E). Fully expanded leaves may retain some of the material, giving the leaf surface a whitish appearance when dry (Fig. 1B).

**Figure 2.**
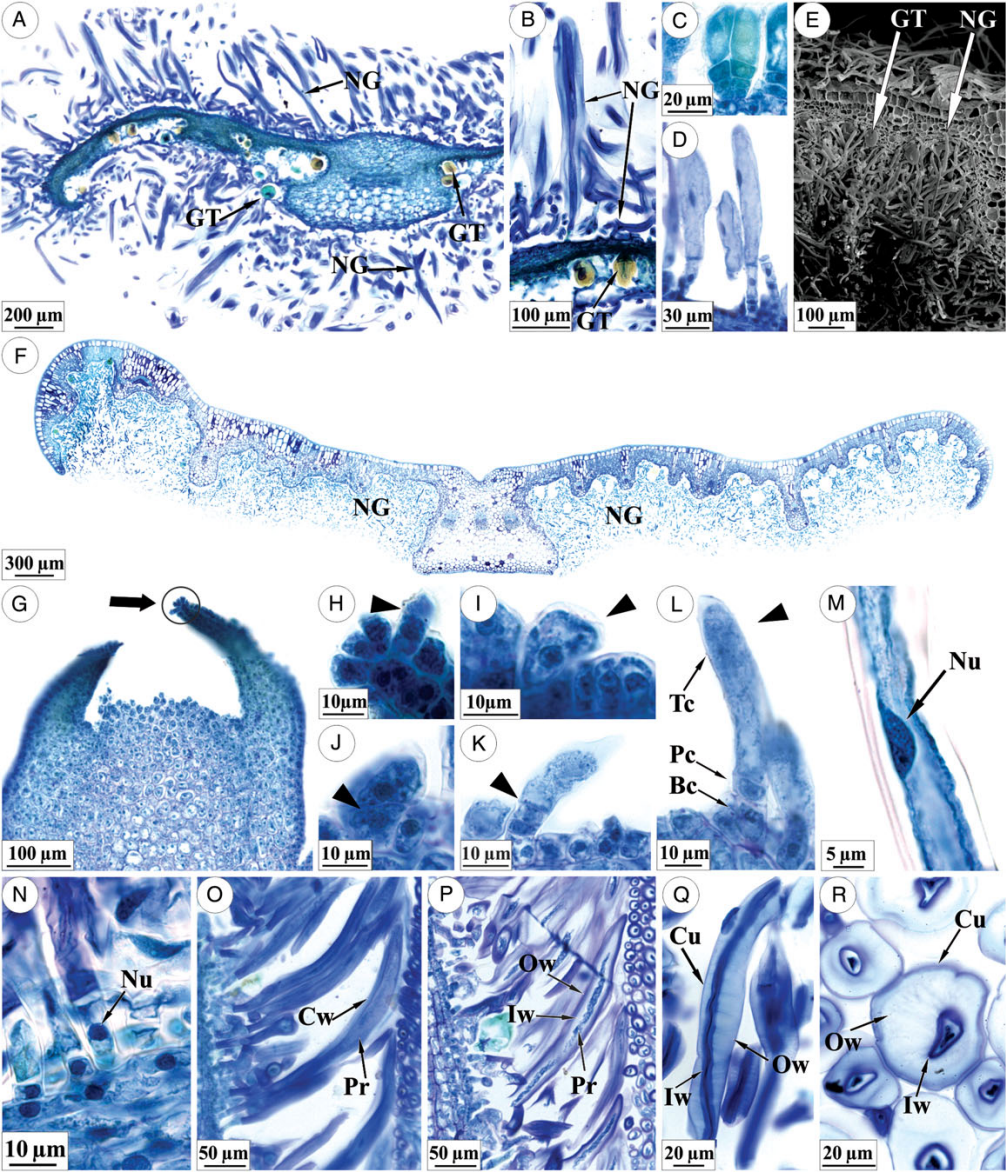
Characterization and development of trichomes in *L. diamantinana*. (A) Cross-section of the leaf primordium displaying non-glandular trichomes (NG) on both surfaces, and glandular trichomes (GT) on the abaxial surface. (B) Details showing an NG with its expanded terminal cell and a GT. (C) Glandular trichome. (D) Early stage of development of NG. (E) Scanning electron micrograph of a cross-section of the young leaf with CTs on the abaxial surface and fibrous NGs. (F) Cross-section of a fully expanded leaf, which is glabrous on the adaxial surface and pilose on the abaxial surface. (G and H) Leaf primordium that exhibits a trichome on its apex (circle in G) showing the beginning of NG differentiation (detail in H). (I–L) Non-glandular development (arrowhead). In (L), note the trichome structure: basal cell (Bc), peduncle cell (Pc) and terminal cell (Tc). (M–P) Non-glandular trichomes with changes in the nucleus (Nu), cell wall (Cw) and protoplast (Pr) of the terminal cell; inner portion of the cell wall (Iw) and outer cell wall (Ow). (Q and R) Terminal cell of the NG trichome sectioned longitudinally (Q) and transversally (R); the images show extended cuticle (Cu), the outer cell wall (Ow) with a loose structure and a compact inner cell wall (Iw).

Leaf primordia and young leaves have two types of trichomes (Fig. 2A and B): glandular trichomes, which have a pair of cells forming the peduncle and four to five pairs of secretory cells (Fig. 2C), and non-glandular trichomes, which are formed by three to seven cells and have an expanded terminal cell that can be simple or branched (Fig. 2B and D). The glandular trichomes are found on the abaxial side of the organ, whereas the non-glandular trichomes occur on both surfaces of the organ (Fig. 2A); the latter form a dense network covering the glandular trichomes, particularly the abaxial surface (Fig. 2E). The adaxial surface of the fully expanded leaves becomes glabrous, while the abaxial side remains pilose (Fig. 2F).

Trichomes differentiate very early, in 0.5-mm-long leaf primordia (Fig. 2G and H). The non-glandular trichomes arise from a protodermal cell with a dense cytoplasm and a conspicuous nucleus (Fig. 2H and I); this cell elongates anticlinally and divides periclinally to form two cells (Fig. 2J). The cell facing the organ becomes the basal cell of the trichome, while the cell facing the exterior divides periclinally to generate between two and six cells (Fig. 2K) and the distal cell expands directly after the last division (Fig. 2L). One or more cells will form the peduncle (Fig. 2L and D). Next, the terminal cell nucleus elongates (Fig. 2D and M) and breaks down, while the other trichome cells remain active and have intact nuclei (Fig. 2N).

In leaf primordia that are ∼2.5-mm long, modifications occur in the cell wall of the terminal cell of the non-glandular trichomes of the young leaves; these modifications include loosening of the cell-wall structure together with retraction of the protoplasm which becomes denser (Fig. 2O–R).

**Figure 3.**
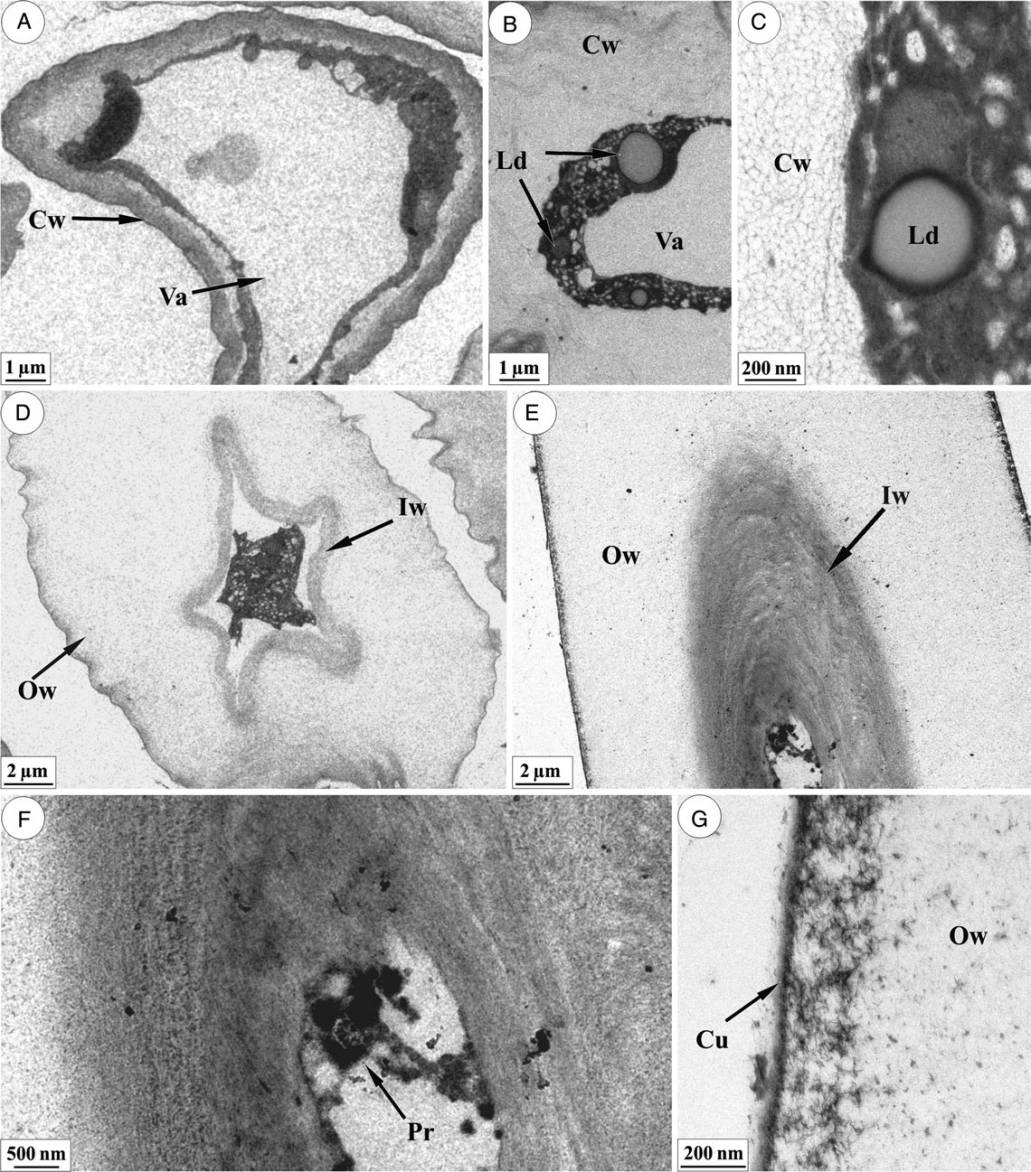
Non-glandular trichomes in leaf primordia of *L. diamantinana.* Transmission electron micrograph of the terminal cell of the trichome. (A) Terminal cell showing a large vacuole (Va) and a slightly cohesive cell wall (Cw). (B and C) Terminal cell exhibiting a cell wall with a very loose structure and a protoplast with lipid droplets (Ld). The fibrillar structure of the cell wall is noted in (C). (D) Overview of a terminal cell with a compact inner portion of the cell wall (Iw) and a loosened outer portion (Ow). (E and F) Inner portion of the cell wall with a compact structure and modified protoplast (Pr). (G) Details of the loosely arranged cuticle and of the outer portion of the cell wall.

The terminal cell of the non-glandular trichome exhibits a well-developed vacuole (Fig. 3A) and lipid droplets dispersed into the cytoplasm (Fig. 3B and C). Parietal degradation begins with the loosening of cellulose microfibrils (Fig. 3C). The inner portion becomes compact (Fig. 3D and F) and the expansion of the pectin matrix occurs in the outer most cell wall areas (Fig. 3D and E). In the cells where the outer part of the wall has degraded, the protoplast is very electron dense and fragmented, with no visible organelles (Fig. 3D and F), and the cuticle loses its cohesive appearance (Fig. 3G).

**Figure 4.**
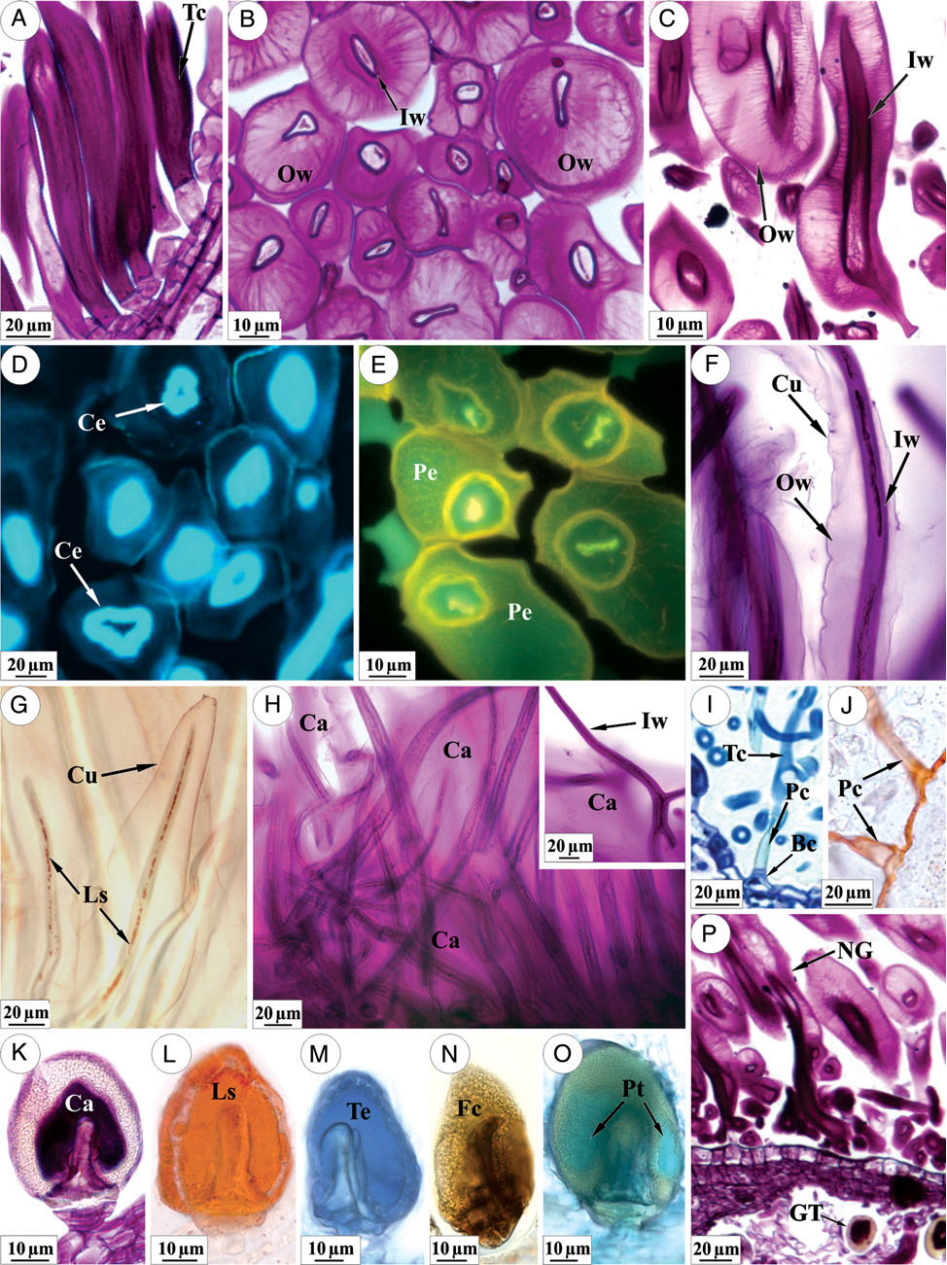
Non-glandular trichomes (NG) (A–J and P) and GT (K–P) in *L. diamantinana.* (A–C) Terminal cells (Tc) displaying carbohydrates in the cell wall. In (A), trichomes before the degradation of outer cell wall. Note in (B)–(C) the compact inner portions of the cell wall (Iw) and the outer portion (Ow) with a loose structure. (D) Cellulose (Ce) in the inner portion of the terminal cell. (E) Pectin (Pe) in the altered cell wall. (F and G) Terminal cell before rupture of the cuticle (Cu). The lipophilic substances (Ls) in the protoplast are shown in (G). (H) Non-glandular trichomes with carbohydrates (Ca) spread over and between the trichomes; the inset illustrates a branched terminal cell after rupture of the cuticle. (I) Non-glandular trichome in a fully expanded leaf exhibiting the basal cell (Bc), peduncle cell (Pc) and terminal cell (Tc). (J) Details of peduncle cells showing the thickening of the suberin that is continuous with the cuticle. (K–O) Glandular trichomes with positive histochemical staining for exudate in the subcuticular space. (P) Young leaf with a large amount of carbohydrates in NG and comparatively less material in GT. Histochemical staining: carbohydrates (Ca) by the Schiff reaction (A–C, F, H, K, P); cellulose (Ce) by calcofluor white (D); pectins (Pe) by coriphosphine (E); lipophilic substances (Ls) by Sudan IV (G, J, L); terpenoids (Te) by the NADI reaction (M); phenolic compounds (Fc) by ferric chloride (N); and proteins (Pt) with aniline blue black (O).

Histochemical analysis showed that the cell wall of the terminal cell of non-glandular trichomes mainly consists of polysaccharides (Fig. 4A–C). After degradation of the outer portion of the cell wall (Fig. 4B and C) it is observed that the inner portion of the cell wall is compact and presents cellulosic nature (Fig. 4D), while in the outer most portion (Fig. 4E) and in the products of the cell-wall degradation most of the carbohydrates are pectins. The inner portion of the cell wall remains compact, while the outer portion is replaced by products that resulted from its degradation (Fig. 4F). Sudan IV testing confirmed that the droplets observed in the protoplast are lipophilic and indicated the presence of cuticle in the terminal cells (Fig. 4G) prior to the release of the cell-wall degradation product. Cuticle rupture leads to the release of the product (Fig. 4H) and leaves the undegraded cellulosic portion exposed (Fig. 4H, inset). After the outer cell-wall degradation, the terminal cell of the trichome lacks protoplasm. In contrast, the basal cell retains intact protoplasm (Fig. 4I).The cell(s) of the peduncle (Fig. 4I) has suberized walls and the suberization is continuous with the cuticle of the common epidermal cells (Fig. 4J).

Glandular trichomes produce hydrophilic (Fig. 4K) and lipophilic substances (Fig. 4L) such as polysaccharides (Fig. 4K), terpenoids (Fig. 4M), phenolic compounds (Fig. 4N) and proteins (Fig. 4O). Despite this production, the secretion of hydrophilic substances by the glandular trichomes is substantially less than the amount of these substances produced by the partial degradation of the wall of the non-glandular trichomes (Fig. 4P).

## Discussion

Cell-wall degradation occurs in a wide variety of situations: as part of normal processes in the plant lifecycle, such as seed germination, xylem vessel formation and the growth, maturation and abscission of fruit; as part of processes where other organisms, such as fungi and herbivores, break down plant material; or even as important tools in industrial processes (Brett and Waldron 1990). However, this study reports for the first time a process of partial cell-wall degradation of the trichome terminal cells in leaf primordia and young leaves, presumably protecting stem apices against dehydration.

The non-glandular trichomes in *L. diamantinana* resemble typical non-glandular covering trichomes, because they do not present cells with a dense cytoplasm, which is the characteristic of secretory cells (Werker 2000). In these trichomes, the cell wall partially degrades in the leaf primordia, after the elongation of the terminal cells is complete, and simultaneously with changes in the protoplast that lead to cell death. A similar cell-wall degradation and programmed cell death process was described by Gunawardena *et al.* (2007) for the leaf development in *Aponogeton madagascariensis* (Aponogetonaceae), where the degradation of the cell wall forms perforations during leaf expansion. The authors observed that the components of the cell matrix are degraded, thus exposing a loose fibrillar network, which is sufficiently weakened to allow mechanical rupture. In the non-glandular trichomes from *L. diamantinana*, the cell-wall degradation products will be released after the cuticle rupture and then deposited on the surface of the developing leaves in the stem apices.

Our study found that the hyaline substance deposited on the stem apices largely consists of pectic cell-wall carbohydrates. The viscosity and gelation properties of a pectin-containing solution are directly related to its chemical composition and structure because the pectin gels form a three-dimensional crystalline network where the water molecules and their co-solutes are captured and show maximum coalescence (Löfgren and Hermansson 2007; Paiva *et al.* 2009). The secretion of hydrophilic compounds in plants is associated with the presence of colleters, i.e. trichomes or protrusions that secrete a sticky substance containing a mixture of mucilage or lipophilic substances that cover apical buds to prevent the desiccation (Fahn 1979). We propose that the hyaline substance observed in *L. diamantinana* has a function analogous to that of colleter secretions based on the following: the similar predominantly hydrophilic composition; the early differentiation of the non-glandular trichomes, which has also been observed in colleters (Appezzato-da-Glória and Estelita 2000; Paiva and Machado 2006; Mayer *et al.* 2011, 2013; Machado *et al.* 2013); and the brief functional time of these structures, which coincides with the initial developmental phases of the leaf or other apical organs when protection against desiccation is essential because these organs are highly susceptible to dehydration (Appezzato-da-Glória and Estelita 2000; Paiva and Machado 2006; Paiva 2009; Mayer *et al.* 2013).

According to Paiva (2009), the presence of hydrophilic material, which is produced by colleters, on the young leaves helps to reduce the amount of water that is lost to the external environment and helps to maintain adequate moisture levels in the developing leaf. These actions continue until the leaves develop other types of protection against desiccation, such as forming a thick cuticle. Hygroscopic polysaccharides that are arranged over the cuticle can improve water retention and promote water vapour absorption through the cuticle (Chamel *et al.* 1991). Furthermore, Miguel *et al.* (2006) and Mayer *et al.* (2013) also suggested that the exudates produced by colleters could act as a physical barrier, protecting the young organs against dehydration. These functions are particularly important for development of the stem apical structures in *L. diamantinana*, which inhabits the ‘campos rupestres’. This environment is characterized by intense solar radiation, water scarcity and fires, especially during the dry season (Ribeiro and Walter 2008); these conditions require increased protection against dehydration. Fog can be found where this species is located, particularly on winter mornings. Thus, the presence of hygroscopic polysaccharides on the stem apices increases the moisture retention under foggy conditions.

Several species that inhabit ‘campos rupestres’ are known by the presence of colleters that provide hydrophilic material, such as species of Apocynaceae (Sales *et al.* 2006; Watanabe *et al.* 2009; Morokawa *et al.* 2013) and Leguminosae (Coutinho *et al.* 2013). Additionally, colleters are related in different families from all over the Cerrado domain that is characterized by fires, nutrient-poor soils, high temperatures and usually with a low water supply (Ratter *et al.* 1997; Ribeiro and Walter 2008; Simon *et al.* 2009). In this domain, the colleters are known in other species of Apocynaceae (Appezzato-da-Glória and Estelita 2000) and in Celastraceae (Mercadante-Simões and Paiva 2013), Leguminosae (Fabaceae) (Paiva and Machado 2006; Paiva 2009) and Rubiaceae (Barreiro and Machado 2007). In all these cases, the occurrence of hygroscopic polysaccharides on young organs probably plays an important protective function.

As reported in this study, in the young leaves of stem apices of *L. diamantinana* were observed glandular trichomes secreting phenolic compounds, lipophilic substances (including terpenoids) and proteins. It is well known that the glandular trichomes are isolated biosynthetically and structurally and for this reason it is probable that they produce large amount of phytotoxic substances that internal leaf tissues could not synthesize (Werker 2000). In this study, we suggest that besides the non-glandular trichomes supplying mostly carbohydrates and promoting the water retention on apices, the glandular trichomes contribute in the apices protection, mainly with terpenoids, phenolic compounds and proteins, that probably repel herbivore and pathogen attacks. This complex composition was previously registered also in secretions of colleters and it was related to this double protection (Miguel *et al.* 2006; Paiva and Machado 2006).

In addition, the function of the non-glandular trichomes in the stem apices of *L. diamantinana* could not be restricted to protection against desiccation. The fibrous structure of the non-glandular trichomes after the rupture of the apical cell cuticle is similar to the structure reported in *Helichrysum aureonitens* and *Pteronia incana* by Afolayan and Meyer (1995) and Mayekiso *et al.* (2008), respectively. According to Afolayan and Meyer (1995), the fibrous appearance of these trichomes and their distribution on the leaf would suggest a protective role against herbivores and fungal spores that are spread by wind. Furthermore, trichomes with extremely elongated cells may also protect shorter glandular trichomes (Werker 2000). We believe that non-glandular trichomes observed in expanded leaves of *L. diamantinana* probably protect the glandular trichomes, by their disposition, forming fibre meshes over these glands. However, more studies might be able to evaluate the probable function of the fibrous structure remaining from non-glandular trichomes in *L. diamantinana*.

## Conclusions

The data on the composition and brief deposition time of hygroscopic hyaline substance on the apical stem organs allow us to infer that the non-glandular trichomes of leaf primordia in *L. diamantinana* function similarly to colleters by helping to protect the developing organs against dehydration. Furthermore, the glandular trichomes secrete terpenoids, phenolic compounds and proteins, which probably repel herbivore and pathogen attacks.

## Sources of Funding

This work was supported by the National Council for Scientific and Technological Development (CNPq)—grants [Proc. no. 302776/2010-9, 302657/2011-8] and the São Paulo Council for Research (FAPESP)—financial support [Thematic Project Proc. no. 2010/51454-3] and grants for the first author [Proc. no. 2010/02085-5].

## Contributions by the Authors

All the authors contributed to a similar extent overall. Each author agreed to the submitted manuscript.

## Conflicts of Interest Statement

None declared.
